# Middle ear cholesteatoma and facial nerve hypertrophy mimicking schwannoma in Charcot-Marie-Tooth disease: A case report

**DOI:** 10.1097/MD.0000000000040347

**Published:** 2024-11-08

**Authors:** Minho Jang, ChanEui Hong, Jin Woo Choi, Chang-Hee Kim

**Affiliations:** a Department of Otorhinolaryngology-Head and Neck Surgery, Konkuk University Medical Center, Research Institute of Medical Science, Konkuk University School of Medicine, Seoul, Republic of Korea; b Department of Radiology, Konkuk University Medical Center, Research Institute of Medical Science, Konkuk University School of Medicine, Seoul, Republic of Korea

**Keywords:** Charcot-Marie-Tooth disease, cranial nerve hypertrophy, direction-changing positional nystagmus, middle ear cholesteatoma, vertigo

## Abstract

**Rationale:**

Charcot-Marie-Tooth (CMT) disease, a hereditary motor and sensory neuropathy, presents with progressive chronic sensory and distal motor polyneuropathy. While sensorineural hearing loss and vestibular impairment have been documented in CMT patients, concurrent middle ear cholesteatoma and persistent direction-changing positional nystagmus have not.

**Patient concerns:**

This study details a 22-year-old man with CMT1 exhibiting these symptoms.

**Diagnoses:**

A 22-year-old man with CMT1 (*PMP22*, c.319+1G>T mutation) presented with rotatory vertigo, left-sided hearing loss, and aural fullness. Examination revealed middle ear effusion and a whitish mass behind the left tympanic membrane. Audiometry showed mixed hearing loss on the left side. Imaging indicated middle ear cholesteatoma and facial nerve hypertrophy.

**Interventions:**

Surgical removal of the cholesteatoma revealed a dehiscent hypertrophied facial nerve.

**Outcomes:**

Postoperative follow-up showed improved hearing and no recurrence.

**Lessons:**

This case highlights 3 key points: facial nerve hypertrophy in CMT requiring differentiation from schwannoma, the first reported instance of middle ear cholesteatoma in a CMT patient, and vertigo due to acute otitis media complicated by serous labyrinthitis, manifesting as direction-changing positional nystagmus. These findings underscore the need for thorough diagnosis and management in such presentations. This is the first report of concomitant middle ear cholesteatoma in a CMT patient, illustrating the complexity of diagnosis and treatment.

## 1. Introduction

Charcot-Marie-Tooth (CMT) hereditary neuropathy, also known as hereditary motor and sensory neuropathy, is characterized by slowly progressive chronic sensory and distal motor polyneuropathy. CMT disease shows heterogeneous clinical manifestations with several phenotypes, including demyelinating CMT1 with severe reduction in nerve conduction velocity, axonal CMT2 without nerve conduction slowing, and dominant intermediate CMT with mixed features of CMT1 and CMT2.^[1]^ The association between CMT and middle ear pathology, such as otitis media with effusion, has not been extensively documented in primary sources. However, the Eustachian tube dysfunction due to neuropathy affecting the muscles involved in the opening of the Eustachian tube, which may lead to otitis media with effusion, has been suspected.^[2]^ Although sensorineural hearing loss or vestibular impairment has been reported in patients with CMT disease,^[3,4]^ those with concomitant middle ear cholesteatoma and rotatory vertigo showing persistent direction-changing positional nystagmus has not been reported. Here we present a 22-year-old man with CMT1 who visited our emergency department with a chief complaint of rotatory vertigo.

## 2. Case presentation

A 22-year-old man, diagnosed with CMT disease by point mutation of peripheral myelin protein 22 (*PMP22*, c.319+1G>T), visited the emergency room with rotatory vertigo lasting for 24 hours. The vertigo was accompanied by left-sided hearing loss and aural fullness. The vertigo was continuous and aggravated by changes in head position. The patient reported mild left-sided hearing loss since childhood but had not sought medical investigation. The left-sided hearing loss and aural fullness worsened with the onset of vertigo.

**Figure 1.**
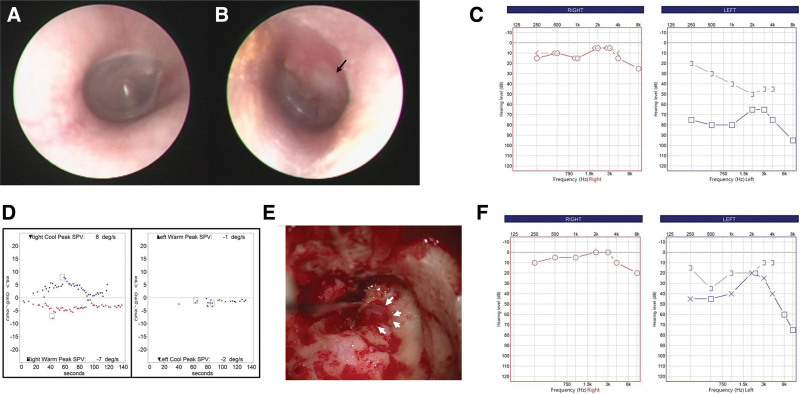
(A) Otoendoscopic examination reveals a normal right tympanic membrane. (B) A whitish mass-like lesion is seen in the postero-superior quadrant of the left tympanic membrane (black arrow). (C) Pure tone audiometry shows normal hearing in the right ear (left panel) and mixed hearing loss in the left ear (right panel). (D) A bithermal caloric test reveals unilateral weakness on the left side. (E) Dehiscence of the hypertrophied facial nerve was observed during surgery (white arrows). (F) posttreatment, left hearing loss improved (right panel).

Otoendoscopic examination revealed a normal right tympanic membrane, while the left side showed middle ear effusion and a whitish mass behind the postero-superior quadrant of the tympanic membrane (Fig. 1A and B). A video frenzel glasses examination showed weakly left-beating spontaneous nystagmus (Video S1, Supplemental Digital Content, http://links.lww.com/MD/N847). Right-beating and left-beating nystagmus were observed in the bow and lean positions, respectively (Video S1, Supplemental Digital Content, http://links.lww.com/MD/N847). A supine head-roll test showed persistent geotropic direction-changing nystagmus, with right-beating nystagmus in the right head-roll and left-beating nystagmus in the left head-roll (Video S1, Supplemental Digital Content, http://links.lww.com/MD/N847). The fistula test was negative. Neurological examinations, including cerebellar function tests and lower cranial nerve examinations, showed no abnormalities. Facial weakness was not observed on either side. Pure tone audiometry revealed no hearing loss on the right side and mixed hearing loss on the left side, with an average bone conduction threshold of 41 dB and air conduction threshold of 73 dB (Fig. 1C).

**Figure 2.**
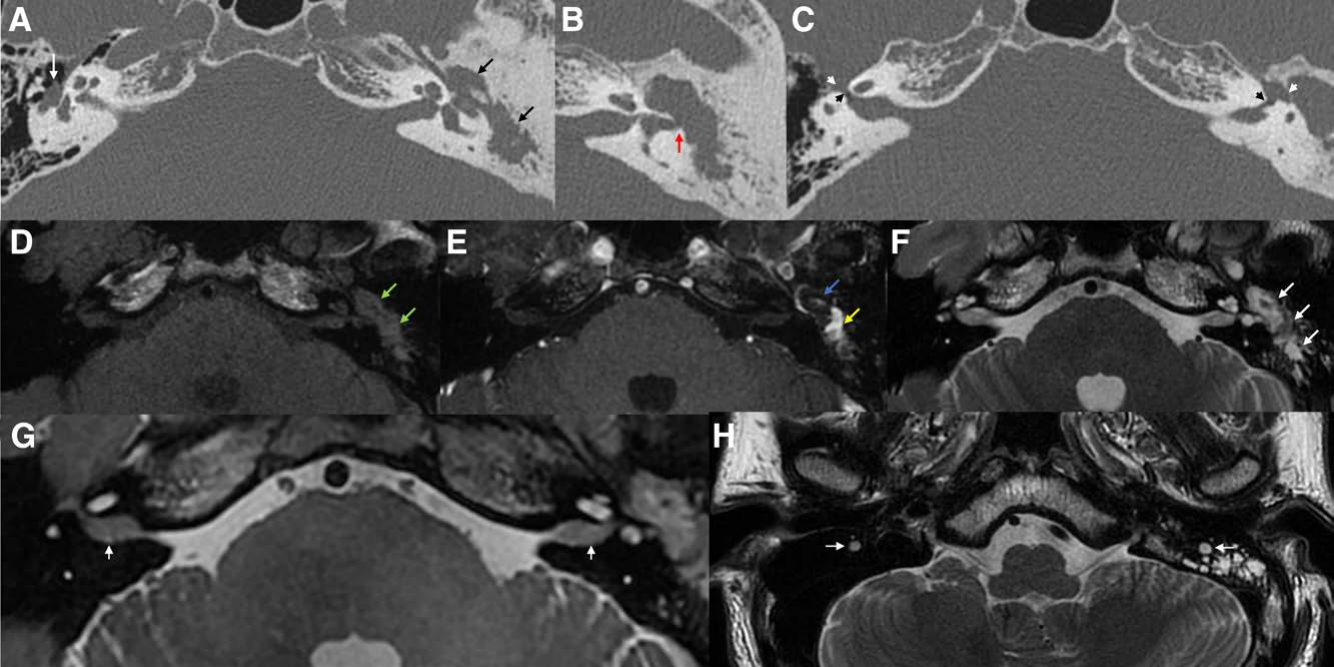
(A–C) Axial view of temporal bone computed tomography. (A) Total opacification of the left middle ear and mastoid cavity (black arrows) with sclerotic changes of the mastoid bone. Prominent soft tissue is noted at the inferior aspect of the mid-tympanic course of the right facial nerve (white arrow). (B) Focal dehiscence of the left lateral semicircular canal was suspected (red arrow). (C) Widening of the bilateral facial nerve canal at the labyrinthine (black arrowheads) and tympanic segment (white arrowheads). (D–F) Axial view of IAC MRI. (D) Precontrast T1-weighted image shows iso-signal intensity lesion in the left middle ear and mastoid cavity (green arrows). (E) Postcontrast T1-weighted image shows enhancing (yellow arrow) and non-enhancing (blue arrow) soft tissue lesion. (F) T2-weighted image shows high signal intensity lesion in the left mastoid and middle ear cavity (white arrows). Axial view of 3-dimensional proton density-weighted image demonstrates enlargement of the canalicular segment (white arrows, G) and mastoid segment (white arrows, H) of bilateral facial nerves.

Following a diagnosis of middle ear cholesteatoma with acute otitis media complicated by serous labyrinthitis on the left side, the patient was admitted for treatment with systemic antibiotics and steroids. Temporal bone computed tomography (TBCT) showed total opacification of the left middle ear cavity and mastoid air cells with sclerotic changes of the mastoid bone. Erosion of the incus long process and stapes suprastructure was observed, and focal dehiscence of the lateral semicircular canal was suspected (Fig. 2A–C). Widening of the bilateral facial nerve canal at the labyrinthine and tympanic segment was noted, with prominent soft tissue at the inferior aspect of the mid-tympanic course of the right facial nerve (Fig. 2A–C), suggesting facial nerve hypertrophy with prolapse into the tympanic cavity or schwannoma. internal auditory canal magnetic resonance imaging (MRI) showed a 1.3 cm non-enhancing T2 high- and T1 iso-signal intensity lesion at the left epitympanum and enhancing T2 high signal intensity lesions in the left mastoid and middle ear cavity, suggesting cholesteatoma (Fig. 2D–F). Enlargement of the bilateral facial nerves was noted in 3-dimensional proton density-weighted images (Fig. 2G and H). Thickening of the V2 and V3 branches of the bilateral trigeminal nerves and the cavernous segments of the bilateral 3rd, 4th and 6th cranial nerves was also observed. A bithermal caloric test showed left unilateral weakness with a canal paresis value of 88% (Fig. 1D).

Under the preoperative diagnosis of the middle ear cholesteatoma with labyrinthine fistula, cholesteatoma removal with canal wall down mastoidectomy was performed. Intraoperatively, a large cholesteatoma sac was observed in the middle ear and mastoid cavity and safely removed. A labyrinthine fistula over the lateral semicircular canal was not observed, and dehiscence of the tympanic segment of the hypertrophied facial nerve was noted (Fig. 1E). The incus long process and stapes suprastructure were eroded, and ossiculoplasty with total ossicular replacement prosthesis was performed. Postoperatively, hearing improved (Fig. 1F) and no evidence of cholesteatoma recurrence was noted during the 2-year follow-up period.

## 3. Discussion

This case presentation raises 3 significant issues worth studying. First, hypertrophy of cranial nerves was observed in our patient with CMT disease, and the facial nerve hypertrophy in preoperative TBCT required differentiation from facial nerve schwannoma during surgery. Second, concomitant middle ear cholesteatoma in a CMT disease patient with facial nerve hypertrophy has not been reported previously. Third, vertigo in this patient might be due to acute otitis media complicated by serous labyrinthitis, with characteristic direction-changing positional nystagmus observed.

Typical clinical features of CMT disease include distal muscle weakness, reduced reflexes, and decreased motor neuron conduction velocity, usually beginning in the second decade. The symptoms are attributed to abnormal neural myelination, with histopathological findings showing onion bulb formations in the involved nerves.^[5]^ One genetic cause of demyelinating CMT (CMT1) disease is defects in the gene encoding myelin protein *PMP22,* as seen in our patient. Different types of *PMP22* gene mutations lead to diverse phenotypes. It has been reported that CMT patients may show progression of sensorineural hearing impairment beyond presbycusis with postnatal onset.^[3]^ In our patient, while the hearing threshold was normal in the right ear, the left ear showed mixed hearing loss, suggesting the hearing loss was caused by middle ear cholesteatoma with effusion and subsequent serous labyrinthitis.

Peripheral nerve hypertrophy is commonly observed in CMT patients, but cranial nerve hypertrophy is rarely reported.^[6–9]^ Since peripheral nerves are in general longer and more susceptible to the effects of the genetic mutations that lead to abnormal myelination than cranial nerves, hypertrophy of the peripheral nerve is more common than cranial nerve. Nerve hypertrophy in CMT is attributed to the accumulation of redundant myelin or Schwann cell hyperplasia, which is prominent in demyelinating type of CMT such as CMT1 which is caused by a mutation of the *PMP22* gene as in our patient. Although cranial nerve hypertrophy in MRI is not pathognomonic for distinguishing CMT from other diseases such as schwannoma, neurolymphoma, and chronic inflammatory demyelinating polyneuropathy, neural hypertrophy in CMT disease tends to show bilateral diffuse enlargement of 1 or more cranial nerves with faint and inconsistent nerve enhancement without leptomeningeal enhancement.^[6–9]^ Johnson et al reported a 34-year-old male with CMT disease who showed markedly asymmetric left more than right facial nerve hypertrophy.^[7]^ L’Heureux-Lebeau et al reported a 28-year-old female with CMT disease presenting with progressive bilateral conductive hearing loss, where the intraoperative mass was the hypertrophic left facial nerve engulfing the ossicular chain with erosion of the stapes.^[8]^ In most case reports, cranial nerve hypertrophy showed poor clinical correlation,^[7–9]^ except for 1 report describing a 64-year-old patient with trigeminal neuralgia and 5th cranial nerve hypertrophy.^[6]^

Our patient presented with rotatory vertigo and left-sided mixed hearing loss, diagnosed with middle ear cholesteatoma with effusion complicated by serous labyrinthitis. Frisch et al reported a 49-year-old polyneuropathy patient with bilateral facial and trigeminal nerve hypertrophy who also had chronic otitis media,^[10]^ postulating that the tympanic segment of the facial nerve was large enough to cause antral blockage, leading to recurrent otomastoiditis.^[10]^ Our patient also had a large dehiscence of the facial canal in the tympanic segment of the hypertrophied facial nerve, which might have caused chronic middle ear inflammation leading to cholesteatoma formation. Considering the normal appearance of the tympanic membrane without perforation and the patient’s history of left-sided hearing loss since childhood, congenital cholesteatoma cannot be ruled out.

Another noteworthy manifestation in our patient was that the direction-changing nystagmus observed with head position changes. Videonystagmography showed persistent geotropic direction-changing positional nystagmus in the supine position, reported in patients with acute otitis media complicated by serous labyrinthitis.^[11,12]^ The underlying mechanism of this characteristic positional nystagmus needs further investigation, but it was postulated that the alterations in the density of inner ear fluids caused by the penetration of bacterial toxin, cytokines, and other mediators from middle ear inflammation into the inner ear fluids through the round or oval window membrane may be responsible for this characteristic positional nystagmus.^[11,12]^

## 4. Conclusion

This study, to our knowledge, is the first report of concomitant middle ear cholesteatoma in a patient with CMT disease. It is noteworthy that mixed hearing loss and vertigo with direction-changing positional nystagmus were observed due to middle ear inflammation complicated by serous labyrinthitis. Multiple cranial nerve hypertrophy, especially dehiscent facial nerve hypertrophy, required careful differentiation from facial nerve schwannoma during surgery.

## Author contributions

**Conceptualization:** Chang-Hee Kim.

**Data curation:** Minho Jang, ChanEui Hong, Jin Woo Choi, Chang-Hee Kim.

**Formal analysis:** Minho Jang, ChanEui Hong, Jin Woo Choi.

**Funding acquisition:** Chang-Hee Kim.

**Investigation:** Minho Jang, ChanEui Hong, Jin Woo Choi, Chang-Hee Kim.

**Project administration:** Chang-Hee Kim.

**Writing – original draft:** Minho Jang, Chang-Hee Kim.

**Writing – review & editing:** Chang-Hee Kim.

## Supplementary Material


